# BAG1: The Guardian of Anti-Apoptotic Proteins in Acute Myeloid Leukemia

**DOI:** 10.1371/journal.pone.0026097

**Published:** 2011-10-10

**Authors:** Sanja Aveic, Martina Pigazzi, Giuseppe Basso

**Affiliations:** Hematology-Oncology Laboratory, Pediatrics Department, University of Padova, Padova, Italy; The University of Birmingham, United Kingdom

## Abstract

BCL2 associated Athano-Gene 1 (BAG1) is a multifunctional protein that has been described to be involved in different cell processes linked to cell survival. It has been reported as deregulated in diverse cancer types. Here, BAG1 protein was found highly expressed in children with acute myeloid leukemia at diagnosis, and in a cohort of leukemic cell lines. A silencing approach was used for determining BAG1's role in AML, finding that its down-regulation decreased expression of BCL2, BCL-XL, MCL1, and phospho-ERK1/2, all proteins able to sustain leukemia, without affecting the pro-apoptotic protein BAX. BAG1 down-regulation was also found to increase expression of BAG3, whose similar activity was able to compensate the loss of function of BAG1. BAG1/BAG3 co-silencing caused an enhanced cell predisposition to death in cell lines and also in primary AML cultures, affecting the same proteins. Cell death was CASPASE-3 dependent, was accompanied by PARP cleavage and documented by an increased release of pro-apoptotic molecules Smac/DIABLO and Cytochrome *c*. BAG1 was found to directly maintain BCL2 and to protect MCL1 from proteasomal degradation by controlling USP9X expression, which appeared to be its novel target. Finally, BAG1 was found able to affect leukemia cell fate by influencing the expression of anti-apoptotic proteins crucial for AML maintenance.

## Introduction

Childhood Acute Myeloid Leukemia (AML) is a clonal disorder characterized by an accumulation of malignant hematopoietic progenitors blocked at various stages of normal myeloid development. Regular proliferation and survival of hematopoietic cells is largely regulated by apoptotic processes which, when severely impaired, could contribute to the pathogenesis of leukemia [1]. The apoptosis-related B cell lymphoma-2 (BCL2) protein family, with its pro-apoptotic (BAX, BAK) and anti-apoptotic (BCL2, MCL1, BCL-XL) members, presents a series of biochemical and cellular events that might be targeted as novel molecular therapeutics [2]. In general, when pro-apoptotic proteins prevail over anti-apoptotic ones, apoptosis occurs [2]–[4]. In AML patients, increased expression of anti-apoptotic BCL2 family proteins has been associated with resistance to chemotherapy, decreased rates of complete remission, and abbreviated survival [5]–[8].

BCL2 associated AthanoGene-1 (BAG1) is a multifunctional protein able to delay cell death by a synergistic action with BCL2 [9]. BAG1 has been described as a part of the regulation of apoptotic, transcriptional, and proliferative pathways, as well as cell signaling and differentiation, although the precise mechanism that lies behind these events has yet to emerge [10]–[12]. Implication of BAG1 in a variety of cellular functions is partially a result of the different subcellular localization of three major BAG1 protein isoforms: nuclear BAG1L (50 kDa), nuclear/cytosolic BAG1M (46 kDa), and cytosolic BAG1 (36 kDa), while the fourth cytosolic isoform of 29 kDa (BAG1S) is less expressed [13], [14]. All isoforms are generated from a single mRNA transcript using alternative translation-start sites [15]. As a result, they share the same C-terminus, but differ in N-terminal extension which, through the ubiquitin-like (UBL) domain, enables interaction between BAG1 and proteasome [16]. The involvement of ubiquitin/proteasome system in the degradation of cellular proteins that regulate different cellular processes, including apoptosis, has been studied widely [17], [18], and BAG1's ability to govern proteasomal degradation of certain proteins, has been already described [19].

BAG1 shares anti-apoptotic features with another family member, BAG3. Both, in fact, have been described to interact with the same partners, such as HSP70 and BCL2, in different models, playing a role in cell survival and maintenance [11], [20]–[22]. Moreover, BAG1 and BAG3 have been involved within the Mitogen-Activated Protein Kinase (MAPK) pathway, principally through c-RAF activation [21], [23], and separate studies confirmed either BAG1 or BAG3 deregulation in diverse cancer types [24]–[28]. In this study, investigation of the potential BAG1 role in AML development was followed. It's protein over-expression was found to maintain the expression of the anti-apoptotic proteins BCL2, BCL-XL, and MCL1. Moreover, BAG1, either directly or indirectly, controls proteasome-dependent protein degradation, extending proliferation and survival of leukemic cells, closely collaborating with its family member BAG3. In addition, the identification of USP9X as a novel BAG1 partner contributes to additional knowledge about AML-evoked processes.

## Results

### BAG1 expression in leukemic patients and leukemic cell lines

**Figure 1:**
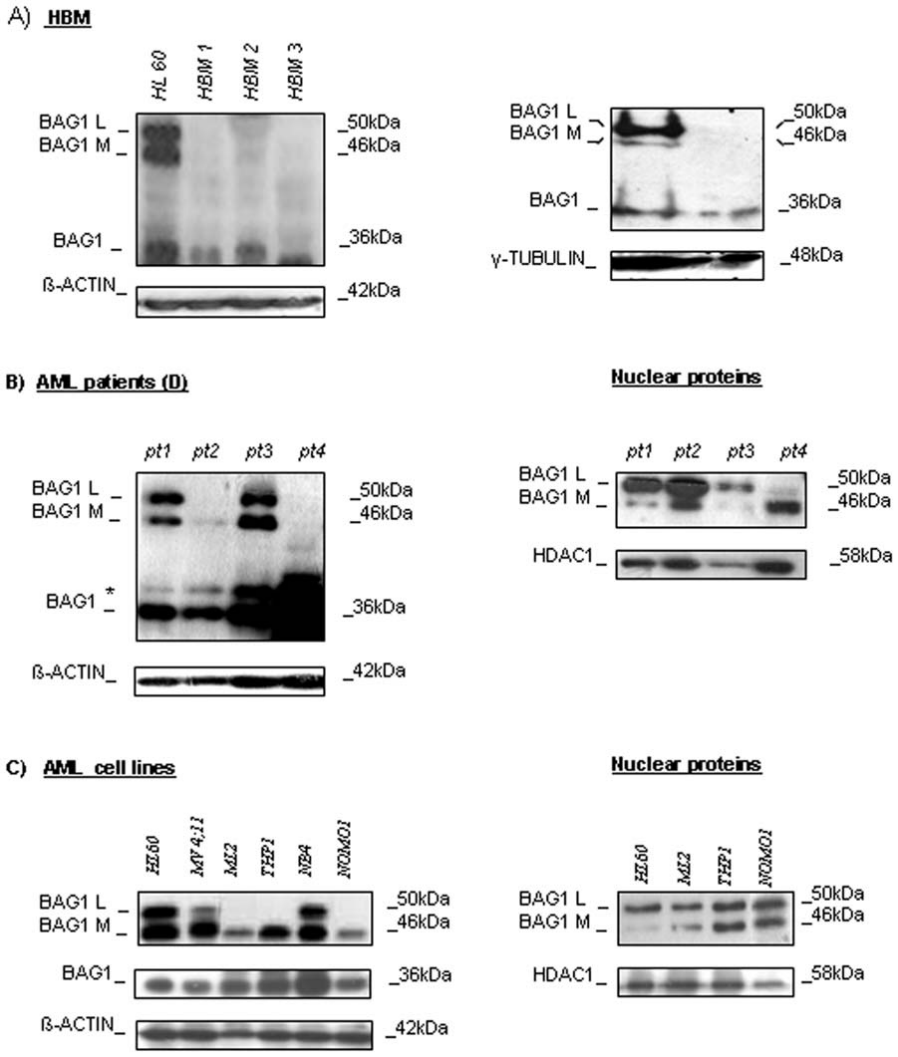
BAG1 protein expression in a cohort of leukemia patients and leukemic cell lines. A) Western Blot (WB) analysis for the study of the expression of three major BAG1 protein isoforms in total protein extracts, and expression of BAG1in CD34+ and CD34- cell fractions sorted from HBM. HL60 cell line is used as positive control B) WB analysis of the expression of BAG1 isoforms in total protein extracts and BAG1L/-1M expression in nuclear proteins isolated from four AML samples at diagnosis (Diag) and from human AML cell lines (C). β-ACTIN is shown as total and HDAC1 as nuclear proteins loading control.

We analyzed the expression of three major BAG1 protein isoforms, BAG1L, -1M, and BAG1, in bone marrow samples from 10 childhood patients with acute leukemia at the moment of diagnosis and compared it with BAG1 expression in 5 healthy bone marrow (HBM) specimens. BAG1L and -1M isoforms were almost undetectable in either total proteins or in proteins from nuclear fractions of HBM, while BAG1 showed faint expression. The same expression profile was observed when CD34+ cell populations isolated from HBM (Figure 1A, Figure S1A). In examined AML patients, with more than 50% of blasts confirmed (Figure 1B: *pt1* = 88%; *pt2* = 53%; *pt3* = 95%; *pt4* = 79%), BAG1 protein was found over-expressed. In particular, BAG1 isoform was always highly expressed, while BAG1L and -1M were expressed variably in total proteins, but were detectable in nuclear protein fractions (Figure 1B). Subsequently, the study of BAG1 protein expression in the cohort of AML cell lines (HL60, NOMO1, NB4, THP1, MV4;11, and ML2) showed BAG1 and -1M isoforms as the mostly abundant and commonly expressed in all examined specimens. BAG1L was not detectable in all of the total protein extracts examined, but, similarly to results obtained for the patients, was confirmed in nuclear protein fractions (Figure 1C). BAG1 mRNA expression was screened in AML patients and cell lines. Results showed large variability demonstrating no evident linear correlation between mRNA and BAG1 protein expression (calibrated with a pool of HBM samples, Figure S1B; not significant).

### BAG1 silencing affected protein expression in HL60 cell line

**Figure 2:**
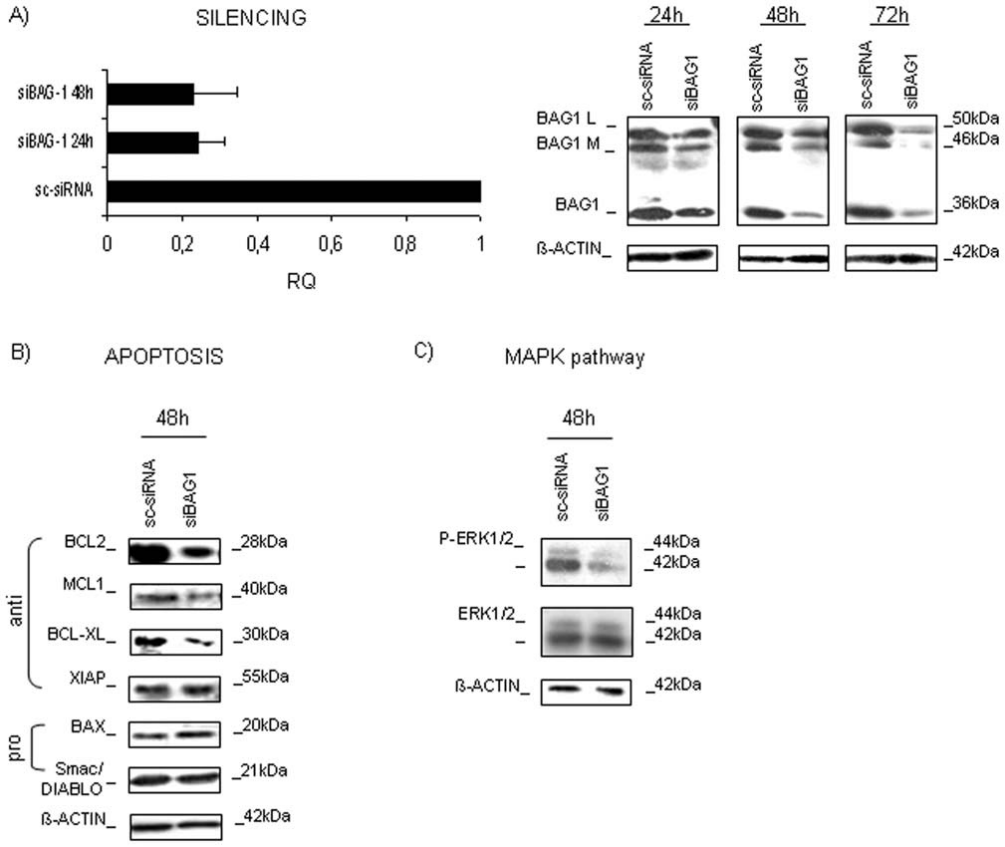
BAG1 silencing in the HL60 cell line. A) BAG1 mRNA and protein expression after silencing are shown. Histogram shows the relative quantification (RQ) of *BAG1* mRNA expression compared to control sc-siRNA silenced HL60 cells (RQ = 1 in the figure; n = 4; **p<0.01). WB analysis confirmed that all three BAG1 protein isoforms were down-regulated after transfection with BAG1 specific siRNA. B) Analysis of pro- and anti-apoptotic proteins expression 48 h post-transfection. C) Expression of MAPK pathway-related proteins validated by WB, 48 h after siBAG1 or sc-siRNA transfection. β-ACTIN is shown as total protein loading control.

**Figure 3:**
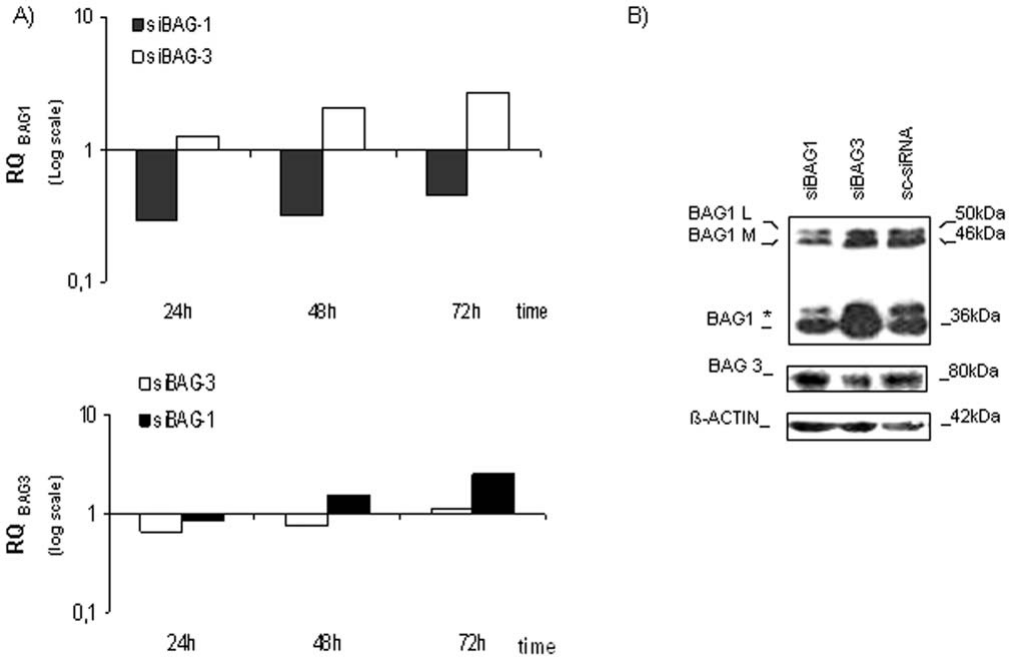
BAG1 and BAG3 mRNA and protein expression in HL60 cells. A) HL60 cells were subjected to silencing of BAG1 (*siBAG1*) and BAG3 (*siBAG3*) and subsequently checked for relative mRNA expression. RQ-values at 24 h, 48 h, and 72 h of transfection are shown after being normalized to *GUS* gene and calibrated to sc-siRNA values. The RQ values are presented on a logarithmic scale. B) WB analysis of BAG1 and BAG3 expression after silencing is presented, as well as the reciprocal protein increase induced after single silencing. β-ACTIN was a control for an equivalent sample loading.

Aberrant BAG1 protein expression in AML suggests it might play a role in leukemia phenotype maintenance. To address this issue, BAG1 was silenced in the HL60 cell line. The silencing resulted in more than 60% of *BAG1* reduction 48 h after transfection at either mRNA or protein level with respect to sc-siRNA (mean RQ_BAG1_ = 0.24±0.05; n = 4; **p<0.01; mean relative expression of BAG1 protein isoforms = 0.45±0.05; n = 3; *p<0.05; Figure 2A). Differences in cell vitality (sc-siRNA vs siBAG1 = 84.9% vs 79.8%) and apoptotic activation (sc-siRNA vs siBAG1 = 10.9±1.7% vs 14.8±2.4%; n = 3; p>0.05) observed between sc-siRNA and siBAG1 transfected samples were not statistically significant (data not shown). Monitoring how protein levels were influenced by BAG1 silencing, we found a notable decrease of BCL2 (0.45±0.15 fold; n = 3; *p<0.05), and also of BCL-XL (0.8±0.2 fold), and MCL1 expression (0.6±0.2 fold; n = 3; p>0.05; Figure 2B). All three proteins were part of the BCL2 family, and all with anti-apoptotic characteristics. No significant changes in expression of BAX, an important pro-apoptotic protein of the BCL2 family, were observed, and same was for XIAP, protein with anti-apoptotic feature, not belonging to BCL2 family, while Smac/DIABLO, protein with pro-apoptotic attribute, increased slightly (1.20±0.25 fold; n = 3; p>0.05; Figure 2B). BAG1 reduction also influenced the MAPK pathway, causing the decrease of the phosphorylated form of the ERK1/2 protein (0.4±0.2; n = 3; *p<0.05) without changing the expression of total ERK (Figure 2C). Measuring the BAX/BCL2 protein index, we found a tendency toward increased expression in siBAG1-silenced samples (BAX/BCL2: sc-siRNA = 1.0±0.2; siBAG1 = 1.6±0.2; n = 3; p>0.05), creating the conditions for apoptosis triggering by reducing the apoptotic threshold. At the mRNA level, BAG1 silencing did not produce substantial changes for either *BCL2*, *MCL1*, or *BAX* (data not shown), whereas *BAG3* mRNA expression was found to increase over time (siBAG1 (24 h, 48 h, 72 h): mean RQ_BAG1_ = (0.29, 0.32, 0.45); mean RQ_BAG3_ = (1.25, 2.08, 2.60); n = 3; *p<0.05; Figure 3A). We confirmed an inverse expression between the two genes, by checking if *BAG3* silencing could affect *BAG1* expression. We confirmed the *BAG1* mRNA and protein increased levels after BAG3 silencing (siBAG3 (24 h, 48 h, 72 h): RQ_BAG3_ = (0.65, 0.76, 1.10); RQ_BAG1_ = (0.85, 1.50, 2.51); n = 3; *p<0.05; Figure 3A,B), as previously seen in another human system by Gamerdinger et al [29]. These results suggest that leukemic cells used BAG3 to compensate the enforced reduction of BAG1, in order to maintain tumor survival.

### BAG1/BAG3 co-silencing significantly harmed HL60 cell line survival

**Figure 4:**
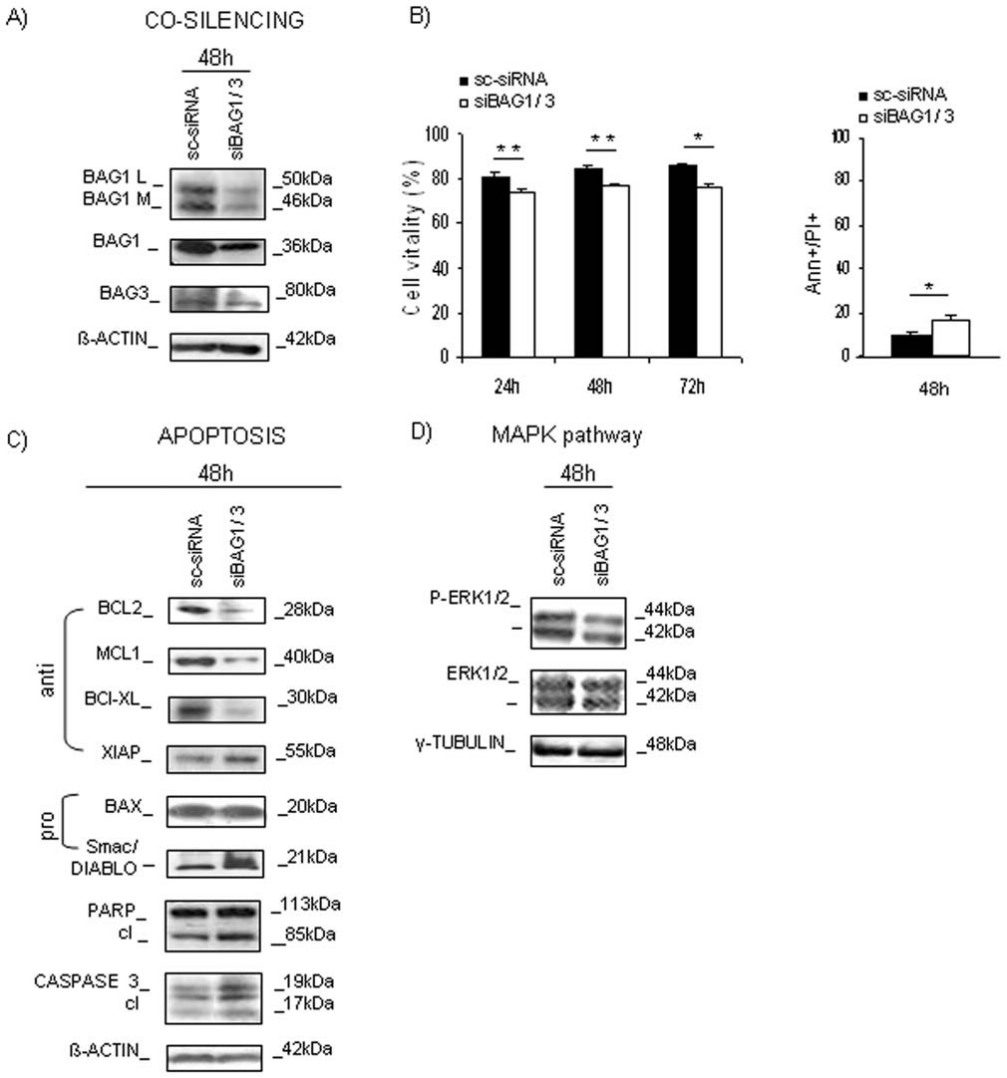
BAG1/3 co-silencing (BCS) in HL60 cells. A) WB after co-silencing is shown at 48 h after transfection. B) Cell vitality was calculated by trypan-blue exclusion assay after BAG1/3 co-silencing (BCS). The first graph shows the percent of living cells 24 h, 48 h, and 72 h after BCS (white bars), or silencing with control sc-siRNA (black bars); the second graph shows the percent of apoptotic (Ann+/PI+) cells 48 h after transfection. Data are presented as mean ± S.E.M. of three independent measurements (n = 3; *p<0.05, **p<0.01). C) The expression of pro- and anti-apoptotic proteins was validated by WB 48 h post-transfection. Additionally, expression of apoptotic hallmarks, CASPASE-3 and PARP, and their cleavage (cl) was examined. D) WB analysis for the expression of MAPK pathway-related protein ERK1/2 after transfection with siBAG1/3 or sc-siRNA. β-ACTIN, and γ-TUBULIN are shown as total protein loading controls.

**Figure 5:**
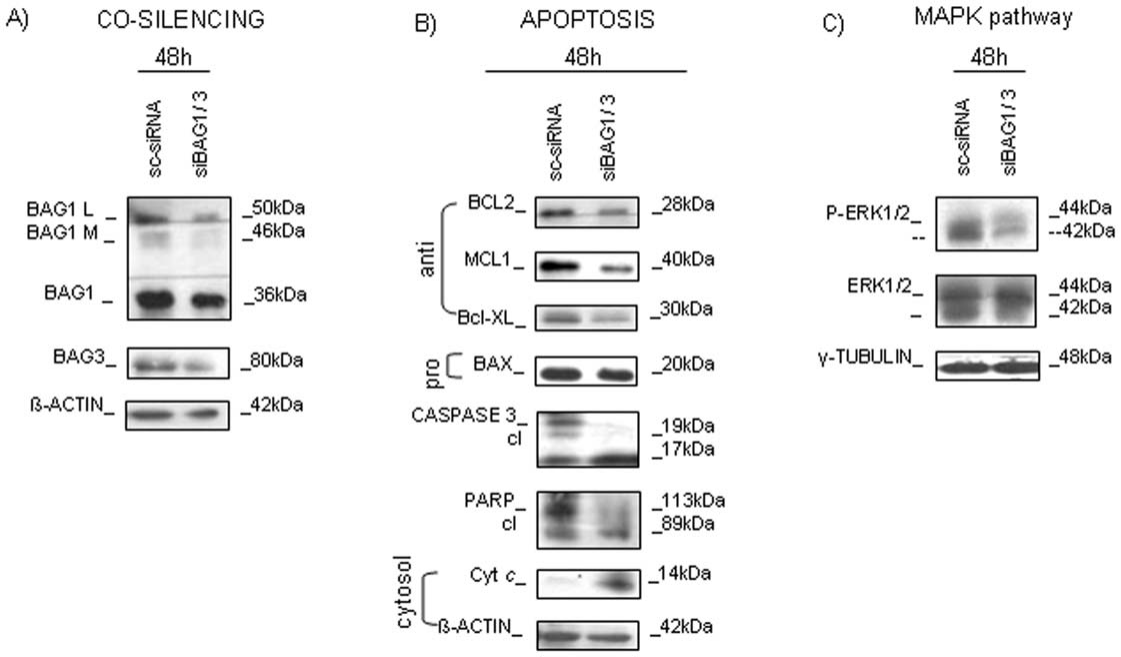
BAG1/3 co-silencing (BCS) in primary AML cultures. A) WB of co-silencing is shown for BAG1 and BAG3 expression after 48 h of transfection B) The expression of pro- and anti-apoptotic proteins was validated by WB 48 h post-transfection. Apoptotic hallmarks, CASPASE-3 and PARP, and their cleavage (cl) was examined. The release of Cyt *c* was evaluated in cytosol protein fraction. C) WB analysis for ERK1/2 expression after transfection with siBAG1/3 or sc-siRNA β-ACTIN, and γ-TUBULIN are shown as total protein loading controls.

The co-silencing of *BAG1* and *BAG3* genes (referred further as BCS) was performed. We confirmed the reduction of their expression at the RNA and protein levels (Figure 4A). After BCS, cell vitality was reduced significantly with respect to sc-siRNA transfected cells (cell vitality (24 h, 48 h, 72 h): mean values for sc-siRNA = (81.4%, 84.3%, 85.0%) and mean values for BCS = (73.7%, 76.5%, 75.8%), respectively; n = 3; *p<0.05; **p<0.01). Moreover, apoptosis induction was also confirmed after 48 h of transfection (Ann+/PI+: sc-siRNA vs BCS = 4.6±1.2% vs 21.1±1.3%; n = 3; *p<0.05; Figure 4B). Protein levels were evaluated by immunoblots after 48 h of silencing because after 72 h of BAG1 silencing, we demonstrated the upregulation of BAG3 that was known to rescue the BAG1 deficiency (Figure 3): as shown in Figure 4C an evident decrease of BCL2 protein expression was verified (0.4±0.1 fold), and of MCL1 (0.5±0.1 fold; n = 3; *p<0.05), and a lower decrease of BCL-XL (0.8±0.2 fold; n = 3; p>0.05), while the expression of the pro-apoptotic molecule BAX was not dramatically changed. The cleavage of the apoptotic hallmark proteins, CASPASE-3 and PARP, was found, and an enhanced expression of Smac/DIABLO was also observed. Phospho-ERK1/2 was found decreased (relative protein expression: sc-siRNA vs BCS = 1.9±0.4 vs 1.0±0.1; n = 4; *p<0.05) while total protein was equally expressed (Figure 4D). The same results in protein impairment were evident after co-silencing of BAG1 and BAG3 in primary AML cultures (Figure 5A, B and C). Cell death with mitochondrial involvement is demonstrated here by a documented Cyt *c* release (Figure 5B). The analysis of eventual changes in mRNA expression of the same genes of impaired proteins was also performed, but results were not informative (data not shown).

### BAG1 and BAG3 co-silencing influenced proteasome-dependent degradation

**Figure 6:**
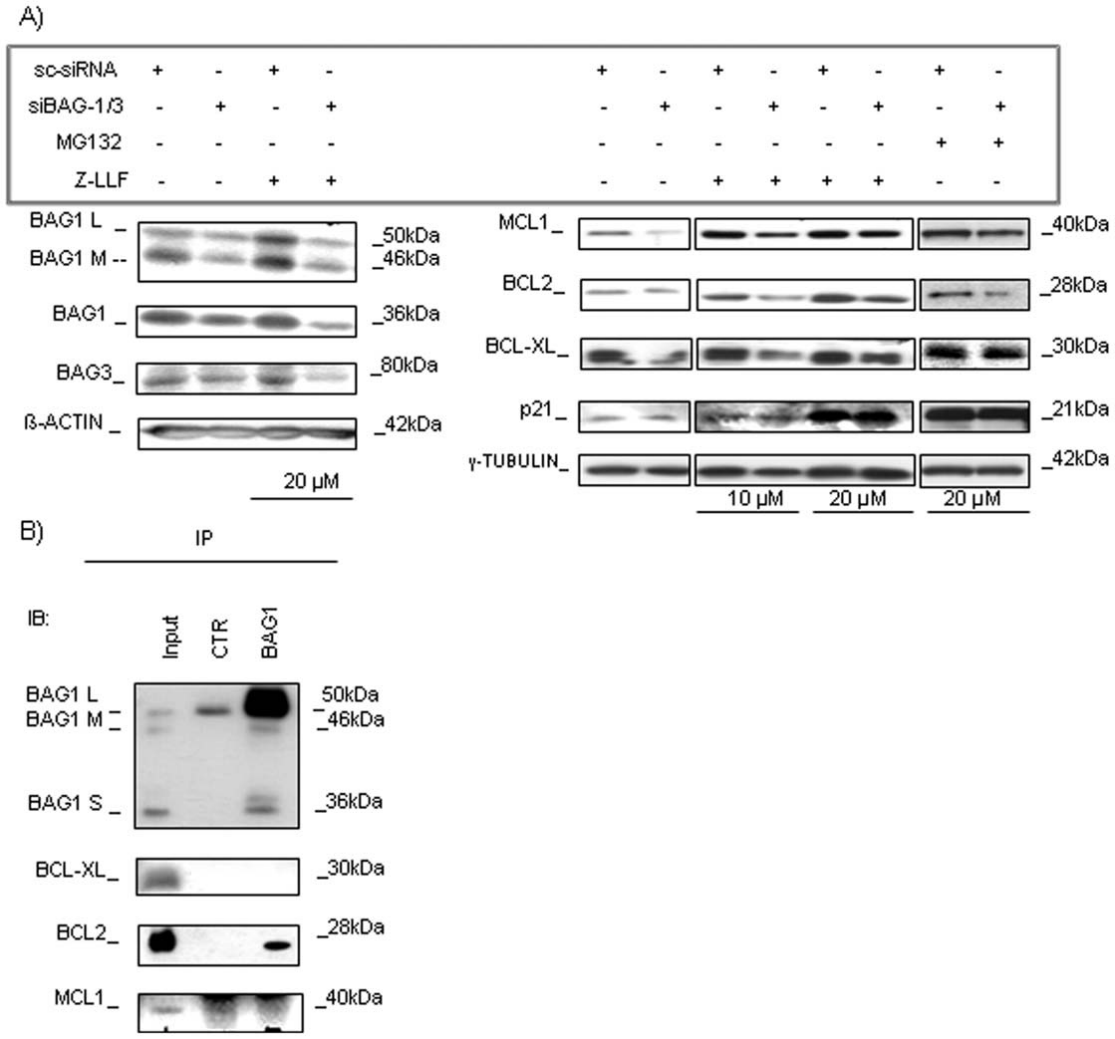
Proteasome inhibition study and co-immunoprecipitation analysis. A) WB analyses after BCS and reversible block of proteasome for 4 h with chemical agents, MG132 (20 µM) and Z-LLF (10 µM and 20 µM), are presented. The eventual changes in BCL2, BCL-XL, and MCL1 protein expression were checked. p21 served as a standard control for proteasome blocking [51], and β-ACTIN and γ-TUBULIN are shown as total protein loading controls. B) Analysis of possible co-interaction between BAG1/BCL-XL, BAG1/BCL2 and BAG1/MCL1 proteins done by immunoprecipitation (IP) and WB is shown.

Since BAG1 interaction with proteasome has been well documented [16], we wished to understand if this pathway is connected to the lowered protein expression described. We blocked proteasome reversibly with the chemical agent Z-LLF and MG132 after BCS to investigate if the BCS enforced proteasome dependent protein degradation. We found similar reproducible results for BAG1 and BAG3, neither of which were significantly influenced by the block of proteasome activity. The sc-siRNA mimics the physiological condition where anti-apoptotic proteins were maintained into leukemic cells to sustain tumor growth. The block of the proteasome increased their expression because a quota of those proteins are continuously degraded by this pathway (2 fold increase protein expression after treatment confirmed by densitometry). After BCS, BCL2, BCL-XL, and MCL1 protein levels were severely decreased, but almost recovered after treatment, suggesting that BAG1 plays a crucial role in preserving these proteins away from degradation. In fact, when proteasome activity was blocked proteins were found into cytosol at the same strength of the sc-siRNA (more evident at 20 µM drug concentration) confirming that the BAG1 expression maintains them away from the proteasome, particularly for MCL1 (by densitometric data: 3 fold recovery increase for BCL-2 for BCS, 6 fold increase for MCL-1, 2-fold for BCL-XL, Figure 6A), suggesting that its over-expression in AML is consistent with a low degradation rate of those proteins. Consistent with a role in mediating the accumulation of the anti-apoptotic proteins in cytosol, we co-immunoprecipitate (co-IP) BAG1 and confirmed a direct interaction between it and the main functional apoptotic target identified here, BCL2. On the contrary, neither BCL-XL nor MCL1 were direct BAG1 partners (Figure 6B).

### USP9X, a novel interaction partner of BAG1

**Figure 7:**
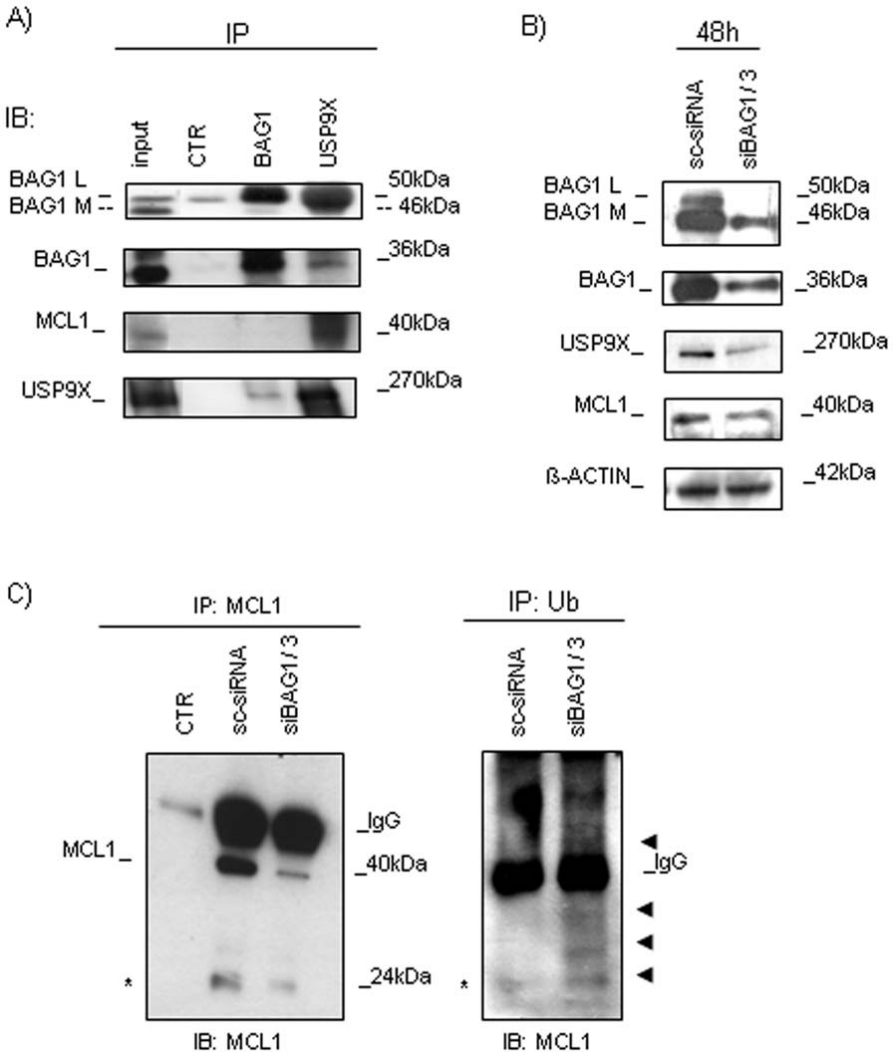
USP9X protein expression and co-immunoprecipitation analysis. A) Analysis of co-interaction between BAG1/USP9X and USP9X/MCL1 proteins was shown. IP assay was done with BAG1 and USP9X primary antibodies and IB with anti-BAG1, anti-USP9X or anti-MCL1 primary antibodies. B) Effects of BCS on USP9X and MCL1 protein expression were analyzed by WB. β-ACTIN is shown as total protein loading control. C) MCL1 was immunoprecipitated after silencing, and the IB analyses with anti-MCL1 and anti-Ubiquitin (Ub) proteins were done. The arrows indicate an increased smear of ubiquitinated proteins after BCS. Asterisk indicates the cleaved form of Mcl-1 [52].

Since MCL1 protein was mainly affected by BAG1 silencing, we looked for other regulatory proteins through which BAG1 could affect its expression. After excluding Aurora-A (data not shown) [30], we found that both, BAG1 and MCL1, immunoprecipitated with USP9X (Figure 7A). This deubiquitinase was shown to control the ubiquitination status of MCL1 protein in AML cells, and here we discovered that USP9X is directly controlled by BAG1. The expression of the USP9X was confirmed to be decreased after BCS (Figure 7B). Hence, the impaired expression of USP9X through BAG1 silencing might affect its interaction with MCL1, creating a conditions for more intensive MCL1 ubiquitination, we immunoblotted MCL1 after silencing BAG1 and found that MCL1 reduced its expression, and moreover increased its ubiquitination (Figure 7C), confirming that BAG1 silencing controls MCL1 expression through USP9X.

## Discussion

It is generally accepted that carcinogenesis is a complicated process that involves many steps for transformation of cells to occur. In childhood Acute Myeloid Leukemia (AML), recent improvements in the outcome have often been related to the presence of specific genetic aberrations [31], [32]. Even if the survival rate is continuously improving, a critical number of patients encounter relapse because they are resistant to current therapies [33]. Therefore, there is a need to understand the molecular pathogenesis of hematopoietic cancers better and to identify new potential molecular targets for treatment.

Altered apoptosis is an important phenomenon responsible for tumorigenesis. BAG1 is a newly discovered anti-apoptotic protein that has been proven to be a multifunctional binding molecule that can enhance the ability of cells to overcome BCL2-mediated protection from apoptosis [34]. The essential role of BAG1 for the survival and differentiation of hematopoietic and neuronal cells has been confirmed by BAG1 knock-out experiments in mice [35]. However, little is known about BAG1 expression and the mechanism of action in leukemic cells of myeloid origin, where other apoptosis-related BCL2 family proteins have been described to be possible molecular therapeutic targets [36].

The first evidence that BAG1 protein is over-expressed in a group of AML patients and leukemic cell lines with respect to healthy donors implied that BAG1 might contribute to leukemia pathogenesis. Additionally, an observed nonlinear correlation between mRNA and BAG1 protein isoform expression levels indicated that a post-transcriptional finely-controlled mechanism might play a crucial role in dictating the final expression of each BAG1 protein isoform in AML.

BAG1 has recently been found implicated in the field of leukemia. It was shown to direct CHIP/Hsc70-regulated protein triage decisions on BCR-ABL immediately after translation to the degradation pathway [37]. Silencing of BAG1 in our AML model was shown to considerably reduce the expression of BCL2, a protein known to be an essential anti-apoptotic effector with important implications in drug resistance, but also of MCL1 and BCL-XL, whose involvement in AML has been well documented [38]–[40]. The observed impairment of the three key anti-apoptotic molecules conferred to BAG1 a crucial role in the control of apoptosis in AML. BAG1 silencing affected significantly the MAPK-pathway, specifically through down-regulation of ERK1/2 activity, upholding this proliferative pathway to be in part maintained by BAG1, as described in other cell types [41]. Despite these findings, only a slight decrease in cell vitality and insignificant apoptosis activation was observed, indicating that leukemic cells were able to successfully over-come the transient reduction of BAG1 expression. The observation that *BAG3* mRNA and protein levels were up-regulated after BAG1 silencing revealed that BAG3, structurally and functionally similar to BAG1, was induced to compensate for BAG1 deprivation, and to maintain a sort of survival rescue of the leukemic cells. A similar functional compensation was previously reported for these two BAG members in other systems [29]. A significant impact on cell behavior was then induced by co-silencing both, *BAG1* and *BAG3*, genes. Co-silencing caused death of leukemic cell lines and primary AML cultures. A down-regulation of BCL2 and phospho-ERK1/2, and a more significant reduction in MCL1 protein expression, were found.

The next step was to consider how BAG1 might influence this protein expression impairment that triggered apoptosis in AML. Among two BAG members considered here, BAG1 is preferentially connected with proteasomal machinery, while BAG3 is described to be involved with autophagy [16], [29]. Therefore, we considered the impact of proteasome inhibition on protein expression after BAG1 and BAG3 co-silencing. A rescue from proteasome degradation of the anti-apoptotic proteins was found, confirming that degradation of BCL2, BCL-XL, and MCL1 occurred preferentially in a proteasome-dependent way after down-regulation of BAG proteins [42], [43]. As we confirmed, BCL2 protection can be influenced through a direct interaction with BAG1 protein. Interestingly, we identified the deubiquitinase USP9X responsible for an indirect down-regulation of the pro-survival protein MCL1 and consequently, apoptosis triggering. The USP9X deubiquitinase has recently been reported to regulate the stability of MCL1 [44], an anti-apoptotic protein expressed in many tumors and associated with drug resistance and survival [40]. The reduced USP9X and MCL1 levels were recently found to block BCR-ABL kinase signaling, taking chronic myelogenous leukemia cells to apoptosis [45]. Here, we identified BAG1 as the real culprit of that apoptotic phenomenon, implying a more important role of BAG1 in AML than previously thought.

Most tumor cells are “primed” for apoptosis, containing a sufficient quantity of pro-apoptotic molecules to effectively trigger cell death, but remain protected by highly expressed anti-apoptotic proteins. Apoptosis-related processes have been reported to be significant in AML, connecting the BAX/BCL2 ratio with the clinical outcome of patients with de novo AML, and elevated MCL1 expression with AML relapses [8], [46], [47]. Here, we propose that targeting BAG1 or BAG3 or other specific ubiquitin-regulators, such as USP9X, may emerge as a novel therapeutic approach to inhibit AML by reducing elevated apoptotic threshold.

In conclusion, we describe a supporting role of BAG1 in the proliferation capacities of myeloid cells, where it maintains a close relationship with BAG3. The down-regulation of both genes restricts cell survival by potentiating proteasome-derived degradation of main pro-survival BCL2 family members, directly or indirectly. USP9X, the important regulator of MCL1 expression [44], has emerged as an interacting partner of BAG1. Considering all results, it is likely that BAG1 does not have a direct anti-apoptotic role in AML cells, but rather performs this function by supporting the expression of other pro-survival molecules. However, further studies need to be done to complete a full image of the working mechanism of BAG1 in AML. Investigations of both BAG members included here may help in better defining a potentially effective therapeutic strategy involving silencing of BAGs or antibody-mediated targeting of BAGs, likely favoring the development of more potent cancer therapy.

## Materials and Methods

### Cell lines, primary cell cultures and patient samples

Human AML cell line HL60 (American Type Culture Collection) was cultured in Dulbecco's modified Eagle medium (DMEM; Invitrogen; Carlsbad, CA), supplemented with 10% fetal bovine serum (FBS; Invitrogen), 1 µg/mL of glutamine, and 1 µg/mL of penicillin/streptomycin (GIBCO® - Invitrogen). Human myeloid cell lines NOMO1, NB4, THP1, MV4;11, and ML2 (all from the American Type Culture Collection) were cultured in RPMI 1640 (Invitrogen) supplemented with 10% FBS, 1 µg/mL of glutamine, and 1 µg/mL of penicillin/streptomycin (GIBCO® - Invitrogen). Analysis of the BAG1 RNA expression in bone marrow samples of 10 newly diagnosed cases of pediatric AML was performed, as well as a series of 10 healthy controls (HBM). Diagnosis of leukemia was carried out according to standard morphologic criteria based on immunohistochemical, immunophenotyping, and cytogenetic studies, following AIEOP-2002 AML treatment protocols [48]. In compliance with the Helsinki protocol, informed consent was obtained from parents. Primary cultures of bone marrow from newly diagnosed AML patients were also studied. Cells were maintained in serum-free RPMI medium (Invitrogen) with 1 µg/mL of glutamine and 1 µg/mL of penicillin/streptomycin (GIBCO® - Invitrogen), interleukin-3 (IL-3) and interleukin-6 (IL-6) (20 ng/mL each; Inalco; Milano, Italy), stem cell factor (SCF), FMS-like tyrosine kinase 3 (Flt-3) ligand, and thrombopoietin (TPO) (50 ng/mL each; Inalco) for 24 h and subsequently subjected to transfection as indicated in the Transient transfection assay section.

### RNA isolation and Reverse transcriptase polymerase chain reaction (RT-PCR)

Total cellular RNA from cell lines and patient bone marrow was extracted with TRIzol reagent (Invitrogen). RNA quality was controlled using an Agilent 2100 Bioanalyzer (Agilent Technologies; Tokyo, Japan). Subsequently, 1 µg of total RNA was reversely transcribed using random hexamers and Superscript II (Invitrogen), according to the manufacturer's instructions. The synthesized cDNA was used for PCR analysis. Primers were generated using Primer Express1.0 software (Table S1), and PCR products sequenced for adequate gene origin confirmation (data not shown).

### Real-time quantitative RT-PCR (RQ-PCR)

RQ-PCR was performed on an Applied Biosystems 7900 HT Sequence Detection System using SYBR Green PCR Master Mixture Reagents (Applied Biosystems; Forest City, CA). Experiments were performed minimum as triplicate and used for relative quantity study. Primers used for RQ-PCR analysis are listed in Table S1. All expression values were normalized using expression of GUS as an endogenous control [49]. For a certain genes (BCL2; BAX), expression after silencing was determined with a human low density immune array profile (Applied Biosystems), and results were interpreted by the comparative ΔΔCt method [50].

### Transient transfection assay

Transfection experiments were performed on HL60 and primary cell cultures. For a single BAG1 silencing, increasing amounts (0.05–0.4 nmol) of specific exogenous small interfering RNAs (siRNA; Santa Cruz (SC) Biotechnology, Santa Cruz, CA) were initially tested, confirming a ratio between 0.15–0.2 nmol as optimal for adequate silencing result. In co-silencing experiments, cells were transfected with specifically recognizing BAG1 (0.15 nmol) and BAG3 (0.2 nmol) siRNA (SC Biotechnology) using Q-017 (HL60) or U-015 (primary cultures) programs from Amaxa Nucleofactor system (Lonza; Cologne, Germany). Applied amounts of siRNA were chosen as optimal for co-silencing, after testing diverse combination, giving acceptable down-regulation efficiency of approximately 70% and avoiding toxic effects on the cells. In both cases, corresponding amount of non-silencing scramble siRNA (sc-siRNA; SC Biotechnology) was used as a control. After transfection, cells were maintained in DMEM (HL60) or RPMI, supplemented with growth factors (primary AML cultures) as described above and with 10% of FBS.

### Apoptosis assay

Cell death was quantified using fluorescein labeled Annexin-V/Propidium Iodide (Ann/PI; Immunostep S.L; Salamanca, Spain). The experimental results were analyzed by a Cytomics FC500 (Beckman Coulter; Brea, CA) for a minimum of 10,000 events.

### Preparation of total protein extracts and nuclear protein fraction

Cells were collected and resuspended in lysis buffer (Biosource International; Camarillo, CA), supplemented with 0.5 mM PhenylMethaneSulfonyl Fluoride (PMSF, Sigma-Aldrich), mammalian protease, and phosphatase inhibitor mixture (both 1×, Sigma-Aldrich), for 30 min on ice for total protein isolation. For the cytosol and nuclear protein isolation, a combination of low salt buffer A (10 mM HEPES pH = 7.9; 1.5 mM MgCl2; 10 mM KCL; and 0.5 mM DTT) and high salt buffer C (20 mM HEPES pH = 7.9; 25% v/v glycerol; 0.42 M NaCl; 1.5 mM MgCl2; 0.2 mM EDTA; and 0.5 mM DTT) was used. Briefly, cell pellets were resuspended in cold buffer A, kept 10 min on ice, and centrifuged at 13,000 rpm for 10 min at 4°C. Supernatants with the cytosol protein fractions were separated, and pellets were gently washed once with cold 1× PBS and re-resuspended in buffer C. Thirty minutes later, the nuclear protein fraction was collected by centrifugation at 13,000 rpm for 30 min at 4°C. Total proteins or proteins from cytosol or nuclear fractions were immediately quantified with a BCA Protein Assay Kit (Pierce; Rockford, IL).

### Western blotting

Aliquots of total or nuclear lysates were subjected to sodium dodecyl sulfate–polyacrylamide gel electrophoresis (SDS-PAGE). Immunoblots were hybridized with the following primary antibodies: BAG1 (recognizing all three BAG1 isoforms), BAG3, Ub (SC Biotechnology), total and Phospho-ERK1/2, CASPASE-3, PARP, BCL2, MCL1, BCL-XL, BAX, Smac/DIABLO, Cytochrome c (Cyt c), XIAP, Aurora A (Cell Signaling; Danvers, MA), and USP9X (gift of prof. Piccolo S. and commercial from Bethyl Laboratories, Montgomery, TX) in concentrations recommended by the manufacturers. Blots were then stripped and re-probed with β-ACTIN, γ-TUBULIN (Sigma-Aldrich) or HDAC1 (SC Biotechnology) antibodies, used as protein loading controls. As secondary antibodies, horseradish peroxidase (HRP)–conjugated anti-rabbit, anti-mouse, or anti-goat IgG (Upstate Biotechnology; Lake Placid, NY) were used. Enhanced chemiluminescence (ECL) Western blotting detection reagents and films (GE Healthcare; UK) were used for protein band acquisition and, when necessary, densitometry was done using ImageJ 1.37v software (National Institutes of Health; Bethesda; MD) and expression of β-ACTIN or γ-TUBULIN for data normalization.

### Co-Immunoprecipitation

For immunoprecipitation (IP) analysis, HL60 and primary AML cells were harvested, washed once in cold 1× PBS, and resuspended in IP buffer (5 mM MgCl2, 137 mM KCl, 1 mM EDTA, 1 mM EGTA, and 20 mM Tris-HCl, pH = 7.5 and 1% CHAPS) containing PMSF, protease, and phosphatase inhibitors (1×; Sigma-Aldrich). After 30 min of incubation on ice, the lysate was clarified by centrifugation, and the supernatant with total protein fraction was pre-cleared with Protein A/G MicroBeads (Miltenyi Biotec, B. Gladbach, Germany) on ice for another 30 min. The lysate was passed over a μ-Column and collected for immunolabeling with anti-BAG1, anti-Aurora A, anti-MCL1 and anti-BCL-XL antibodies for 30 min on ice (SC Biotechnology). For immunolabeling with anti-USP9X, and anti-MCL1 and anti-Ub after BCS, small modification of protocol was introduced, considering the incubation with MicroBeads over night with 500 µg of total proteins. Subsequently, lysates retaining magnetically labeled protein were run through the μ-Column, and after four washes, eluted with pre-heated (95°C) 1× SDS loading buffer. The immunoprecipitates were analyzed by SDS-PAGE.

### Reagents and treatments

Cell viability was determined by trypan-blue (GIBCO® - Invitrogen) exclusion assay, counting by phase-contrast microscopy. Proteasome inhibition was obtained using a selective and reversible proteasome inhibitors MG132 (20 µM; Sigma-Aldrich) and (benzyloxycarbonyl)-leu-leu-phenylalaninal (Z-LLF-CHO; 10 µM and 20 µM; Sigma-Aldrich) 48 h post-transfection for 4 hours.

### Statistical analysis

Statistical analysis was performed using Prism 4.02 (Graph Pad Software; La Jolla; CA). All experiments were performed in triplicate, and mean values are presented as mean ± standard error of mean (S.E.M.) of replicate experiments. Statistical significance was evaluated by the unpaired Student's t-test. Differences were considered significant for p<0.05 and presented as *p<0.05; **p<0.01, ***p<0.001.

## Supporting Information

Figure S1 **BAG-1 protein in HBM and mRNA expression in a cohort of AML patients and AML cell lines.** A) Expression of BAG1 protein isoforms within nuclear protein fractions isolated from HBM specimens. HDAC1 is shown as nuclear proteins loading control. B) The *BAG1* mRNA expression was validated for a cohort of AML patients at diagnosis and for the AML cell lines. Histograms show the relative quantification (RQ) of *BAG1* mRNA expression compared to control samples of healthy donors (RQ = 1 on the figure; n = 4; p>0.05). All results are normalized for *GUS* as endogenous control.
(TIF)

Table S1 **Table of primers used for PCR or RQ-PCR analyses.**
(DOC)
