# Supplementary figures and images for: BAG1: The Guardian of Anti-Apoptotic Proteins in Acute Myeloid Leukemia
Source: PLoS One. 2011 Oct 10;6(10):e26097. doi: 10.1371/journal.pone.0026097 (PMC3189928; doi:10.1371/journal.pone.0026097)

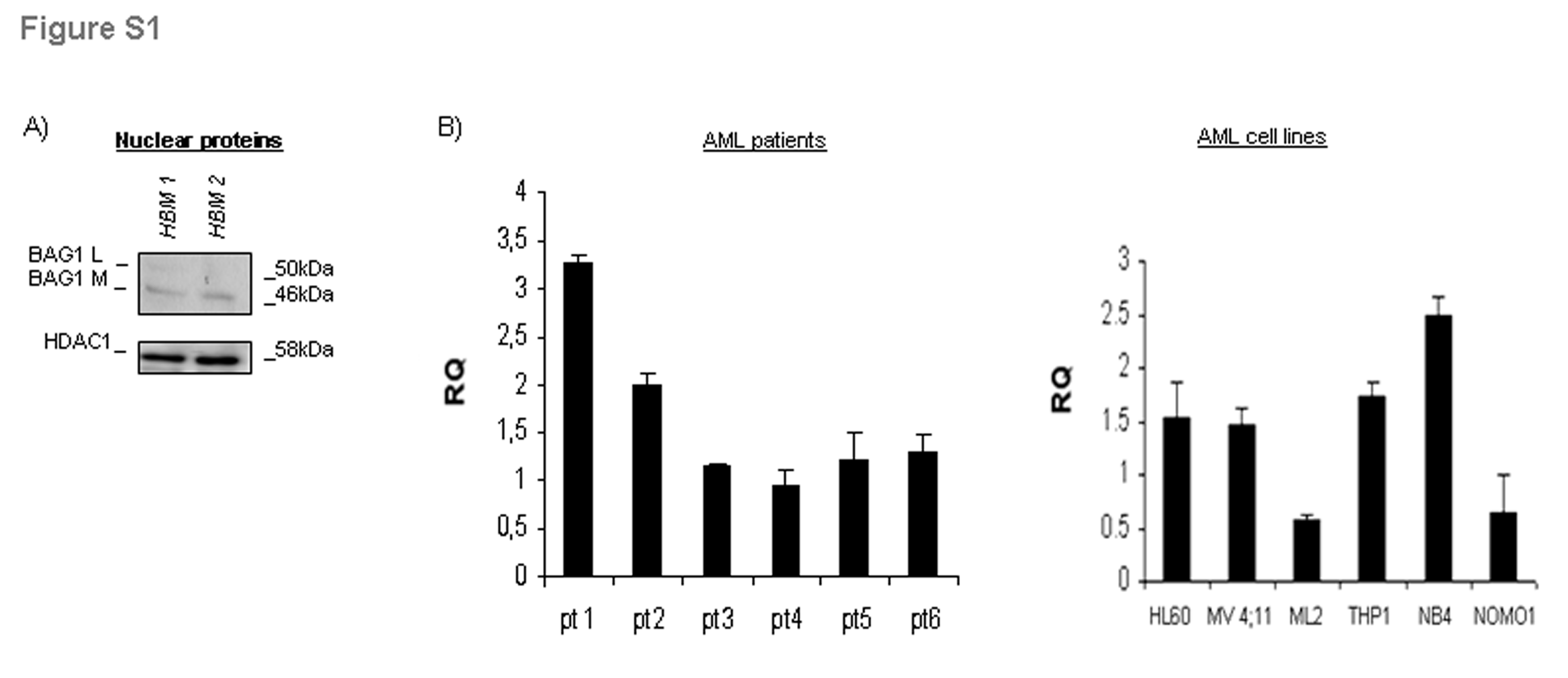

Supplement: Figure S1 — BAG-1 protein in HBM and mRNA expression in a cohort of AML patients and AML cell lines. A) Expression of BAG1 protein isoforms within nuclear protein fractions isolated from HBM specimens. HDAC1 is shown as nuclear proteins loading control. B) The BAG1 mRNA expression was validated for a cohort of AML patients at diagnosis and for the AML cell lines. Histograms show the relative quantification (RQ) of BAG1 mRNA expression compared to control samples of healthy donors (RQ = 1 on the figure; n = 4; p>0.05). All results are normalized for GUS as endogenous control. (TIF) [file pone.0026097.s001.tif]
