# Supplementary material for: BAG1: The Guardian of Anti-Apoptotic Proteins in Acute Myeloid Leukemia
Source: PLoS One. 2011 Oct 10;6(10):e26097. doi: 10.1371/journal.pone.0026097 (PMC3189928; doi:10.1371/journal.pone.0026097)
Supplement: Table S1 — Table of primers used for PCR or RQ-PCR analyses. (DOC) [file pone.0026097.s002.doc]

**Supplementary Table S1.**

| Table S1. **PCR and RQ-PCR primer sequences** | | |
| --- | --- | --- |
| Gene | Primers’ pair sequences | Frag.size |
| **bag-1a** | F: 5’-GGAGCCGCGCCAGTC-3’  R: 5’-CCTGGTGGGTCGGTCATG-3’ | 100bp |
| **bag-1b** | F: 5’-TGACTGTCACCCACAGCAATGA-3’  R: 5’-TCAGTGTGTCAATCTCCTCCAAGA-3’ | 450bp |
| **bag-1c** | F: 5’-CTCCCTCTGGGCGTCCA-3’  R:-5’-TCTCCACAGACTTCTCCAAATGTTT-3’ | 600bp |
| **bag-1d*** | F: 5’-CTCGACCCGGAGCGAGGAG-3’  R: 5’-GCTTCTCATTGCTGTGGGTGA-3’ | 186bp |
| **bag-3*** | F: 5’-CAGCCAGATAAACAGTGTGGAC-3’  R: 5’-AGAGGCAGCTGGAGACTGG-3’ | 95bp |
| **gus*** | F: 5’-GAAAATACGTGGTTGGAGAGC-3’  R: 5’-CGAGTGAAGATCCCCTTTTTA-3’ | 99bp |
| **bcl-2*** | F: 5’- AGTACCTGAACCGGCACCT -3’  R: 5’- GGCCGTACAGTTCCACAAA -3’ | 74bp |
| **mcl-1**** | F: 5’-CGACCCCCGCGAGGCTGCTTTTCT-3’  R: 5’-CTGGCGGCGGCGTCGAGGGTAGT-3’ | 227bp |
| **c-abl** | F: 5’-CCTTCTCGCTGGACCCAGTGA-3’  R: 5’-TGTGATTATAGCCTAAGACCCGGAG-3’ | 152bp |

Table of primers used for PCR or RQ-PCR analyses. bag-1a,b,c,d – primers recognizing several positions on bag-1’s gene, *primers used for RQ-PCR analysis; **RQ-PCR pair of primers from Jacobs AT, et al. 2009. Abbreviations: F, forward primer; R, reverse primer; Frag.size, fragment size; bp, base pairs.
